# A novel cuproptosis-related prognostic lncRNA signature for predicting immune and drug therapy response in hepatocellular carcinoma

**DOI:** 10.3389/fimmu.2022.954653

**Published:** 2022-09-15

**Authors:** Shujia Chen, Peiyan Liu, Lili Zhao, Ping Han, Jie Liu, Hang Yang, Jia Li

**Affiliations:** ^1^ Clinical School of the Second People’s Hospital, Tianjin Medical University, Tianjin, China; ^2^ Department of Hepatology, Tianjin Second People’s Hospital, Tianjin, China

**Keywords:** lncRNAs, immune, drug therapy, hepatocellular carcinoma, cuproptosis

## Abstract

Intratumoral copper levels are closely associated with immune escape from diverse cancers. Cuproptosis-related lncRNAs (CRLs), however, have an unclear relationship with hepatocellular carcinoma (HCC). Gene expression data from 51 normal tissues and 373 liver cancer tissues from the Cancer Genome Atlas (TCGA) database were collected and analyzed. To identify CRLs, we employed differentially expressed protein-coding genes (DE-PCGs)/lncRNAs (DE-lncRNAs) analysis, Kaplan–Meier (K-M) analysis, and univariate regression. By univariate and Lasso Cox regression analyses, we screened 10 prognosis-related lncRNAs. Subsequently, five CRLs were identified by multivariable Cox regression analysis to construct the prognosis model. This feature is an independent prognostic indicator to forecast overall survival. According to Gene Set Variation Analysis (GSVA) and Gene Ontology (GO), both immune-related biological processes (BPS) and pathways have CRL participation. In addition, we found that the characteristics of CRLs were associated with the expression of the tumor microenvironment (TME) and crucial immune checkpoints. CRLs could predict the clinical response to immunotherapy based on the studies of tumor immune dysfunction and rejection (TIDE) analysis. Additionally, it was verified that tumor mutational burden survival and prognosis were greatly different between high-risk and low-risk groups. Finally, we screened potential sensitive drugs for HCC. In conclusion, this study provides insight into the TME status in patients with HCC and lays a basis for immunotherapy and the selection of sensitive drugs.

## Introduction

Hepatocellular carcinoma (HCC) is one of the most frequent malignancies worldwide and is the second dominant cause of cancer-related deaths (1, 2). More than 700,000 people die of liver cancer globally, with approximately 500,000 new cases annually (1). Hepatitis C virus (HCV) infection, chronic hepatitis B virus (HBV), non-alcoholic fatty liver disease, and alcoholic liver disease all contribute to HCC (2). Current therapy options for early-stage HCC include radiofrequency ablation surgery and liver transplantation (3–6). Recurrence, or the occurrence of distant metastases, occurs in most patients after surgery (7). Unfortunately, the diagnosis is advanced in more than 70% of patients. As a result, only restricted therapeutic help is available for a small number of patients. Thus, it is important to elucidate the molecular mechanism of HCC progression and set novel molecular goals for HCC diagnosis and treatment.

One of the fundamental mineral nutrients for all living things is copper (Cu), which is the foundation for many biological activities containing antioxidant/detoxification activities and mitochondrial respiration (8). Recently, cuproptosis has been considered as a copper-triggered mode of mitochondrial cell death (9). Moreover, many links between the disease status and Cu have been observed, and several studies have reported higher copper levels in cancer malignancies than in normal tissues. There is a relationship between copper accumulation and cell propagation, as well as angiogenesis. It can be seen that in cancer, copper imbalance exerts a dominant function. In particular, it has been found that there were great variations in the serum and tumor tissue levels of Cu in patients with diverse cancers such as ovarian cancer, pancreatic cancer, prostate cancer, cervical cancer, breast cancer, gastric cancer, lung cancer, and thyroid cancer (7, 10–18).

Long non-coding RNAs, or lncRNAs, are a family of transcripts of non-coding molecules over 200 nucleotides in length that are thought to exert important functions in diverse diseases (19, 20). Abnormal lncRNA presentation was greatly associated with tumor malignancy, including HCC (21–25). For example, according to previous reports, the lncRNA Miat family promotes the proliferation, invasion, and migration of HCC cells by sponging miR-214 (21); hepatoma cell propagation, migration, and chemoresistance could be virtually suppressed by lncRNA SNHG16 upregulation by functional cavernous hsa-mir-93 (22). Moreover, lncRNA HULC could cause autophagy. For instance, it has been reported that stabilizing SIRT1 lowered the sensitivity of HCC cells to chemotherapeutic drugs (23). However, the role of CRL imbalance in tumor progression is not well defined. Not much research had paid attention to the regulatory relationship between CRLs and HCC. Exploring the relationship between CRLs and HCC development could be useful for recognizing underlying indicators as therapeutic goals.

This paper performed a prognostic feature of lncRNAs related to cuproptosis (LINC01515, AC105020.5, AC019069.1, HCG15, AC079209.1). It is an independent prognostic indicator with high accuracy in forecasting overall survival (OS). This study shows that the characteristic is related to immune-related functional pathways, which exerted crucial function in HCC tumorigenesis, and is closely associated with the tumor microenvironment (TME), immunotherapy, and chemical drug response. Our study constructed a new prognosis model based on CRLs, which provides possible value in the prognosis of HCC patients and provides benefits in guiding individualized immune and drug therapy.

## Materials and methods

### Data sets and patients

From The Cancer Genome Atlas-Liver hepatocellular carcinoma (TCGA-LIHC) database (https://portal.gdc.cancer.gov/) RNA-seq, we collected transcriptome data from 373 HCC samples and 51 normal samples. In addition, we obtained the matching clinical and pathological characteristics, covering tumor grade, age, follow-up time, sex, Tumor, Node, Metastasis (TNM) phrase, and survival condition. We combined profiles from replicate samples from the same patient into an average. We further differentiated the transcriptomic data of TCGA-LIHC from mRNA and lncRNA and collected 19,323 mRNAs and 13,162 lncRNAs in HCC. We used TCGA-LIHC in the University of California Santa Cruz (UCSC) Xena (https://xena.ucsc.edu/) database to extract the copy number variation information of LIHC. As a public database, each case involved in TCGA has gained ethical agreement and is approved by TCGA. Individual researchers analyzed the database. Open-source data were the foundation of this work. We blinded related identifying data for all included cases, making the study plan ethical. We proceeded and reported this study based on the Declaration of Helsinki. **Supplementary Figure 1** shows the data analysis process.

### Identification of cuproptosis-related lncRNAs with prognostic significance in hepatocellular carcinoma

We first reviewed the literature and summarized the cuproptosis-related genes, and obtained a total of 19 genes (see **Table S1**). Next, the expression of cuproptosis-related genes was obtained by the R “limma” package (26). According to the correlation coefficient > 0.3, *P*< 0.05, 53 CRLs and their expression were identified. Finally, the coexpression of cuproptosis-related genes and CRLs were analyzed using the “ggplot2” and “ggalluvial” R package (27) to observe the interaction.

### Construction of the risk score model based on prognostic cuproptosis-related lncRNAs in hepatocellular carcinoma

We collected 10 lncRNAs associated with cuproptosis by univariate Cox analysis. We employed Lasso Cox regression and applied the R package “glmnet” to remove highly correlated lncRNAs (28). Finally, this study recognized only five CRLs and entered into a fresh risk-scoring model. According to the prediction model, the CRLs of each HCC patient can be derived from the formula below:


CI (cuproptosisindex)=ΣExpi * βi

(βi stands for each lncRNA coefficient, and Expi stands for each lncRNA presentation).

Next, we randomly divided all patients into two sets (184 in the training group and 182 in the testing group). Patients were split into high-risk and low-risk groups based on risk scores within each cohort. We contrasted CRL median cutoff values and split patients into high-CRL and low-CRL groups. We used the R package “survival” (29) to perform a Kaplan–Meier (K-M) analysis of OS on the high- and low-CRL groups of the three data sets and evaluated model feasibility (30). Furthermore, through K-M analysis, there was no progression-free survival (PFS) in the training group between the high-CRL group and the low-CRL group.

### A comprehensive evaluation of cuproptosis-related index and clinical parameters in patients with hepatocellular carcinoma

To further clarify the clinical practicality of CRLs, PCA analysis was carried out to prove whether the lncRNA involved in the model construction can distinguish these two groups of patients. In addition, through hierarchical analysis, the correlation between this formed model and many clinical markers containing the grade, gender, age, stage, and T was determined.

### Development and evaluation of clinical pathological nomogram related to cuproptosis

We determined whether CRLs were independent prognostic HCC indicators by using univariate and multivariate Cox regression analyses. The mentioned findings showed “RMS” and the “regplot” R package, and a clinicopathological nomogram related to cuproptosis was exploited (31). We described the decision curve analysis (DCA) of HCC patients and the calibration curve (32) to verify that the nomogram prediction and recognition outcomes were content.

### Functional enrichment analysis of differentially expressed genes related to cuproptosis

We deciphered the majorly enriched signaling pathways and biological roles between the high- and low-CRL groups in the training sets through GO and GSVA (33). *P*< 0.05, with |NES| > 1.5 and FDR q-value< 0.1, was statistically significant.

### Tumor somatic mutation and differential tumor mutational burden and survival analysis

We utilized the “maftools” package (34) to assess and contrast the gene mutation frequencies between the two groups using the tumor somatic mutation waterfall method established in the high-risk and low-risk scores. For mutation type analysis, we selected the first few genes with high mutation frequencies. Secondly, we applied “limma” and “ggpubr” packages (26) to show the different analyses of survival analysis and tumor mutational burden (TMB) and then compared the prognosis and tumor mutation of the two groups.

### The potential significance of immunotherapy based on characteristics and tumor immune microenvironment landscape estimation

Immunotherapy and targeted therapy techniques for HCC patients in recent years have been continuously improved. Consequently, this study evaluated the correlation between five lncRNAs and immune checkpoints (35) by using the Wilcoxon test and verified its accuracy with the Shapiro–Wilk normality test. We applied the single-sample gene set enrichment analysis (ssGSEA) method to calculate the four tumor immune-infiltrating cells’ enrichment in the gene expression matrix of the TCGA-LIHC cohort in high-CRL and low-CRL HCC samples. The outcomes of immunoassays were represented by scatter plots, and *P*< 0.05 was regarded statistically significant by Spearman’s test. The characteristics of CRLs in HCC and the differences in immune function, immune escape, and immunotherapy were analyzed, and the effect of immunotherapy in high- and low-risk patients was evaluated.

### Screening potential drugs for hepatocellular carcinoma

The “limma” and “ggpubr” packages (26) are used to predict which high-risk and low-risk groups have different susceptibilities to the drug. The filtration condition was *P*< 0.05. The lower the IC50 value, the more sensitive it is to drugs, to guide patients’ clinical medication.

### Statistical analysis

We used R software (version 4.0.2, http://www.R-project.org) to analyze all statistics. We used the log-rank test and compared each K-M curve included. Then, we utilized the Wilcoxon test to examine CRL expression levels in normal and HCC tissues in low- and high-CRL groups. In addition, we stratified the differences in the adjusted CRLs values for each clinicopathological parameter. Additionally, we screened CRLs and OS for independent prognostic indicators associated with OS by univariate and multivariate Cox regression analyses. The Spearman correlation test represented the correlation matrix. *P*< 0.05 was considered statistically significant, and the *P*-value was two sided.

## Results

### Identification and construction of cuproptosis-related lncRNAs

**Figure 1:**
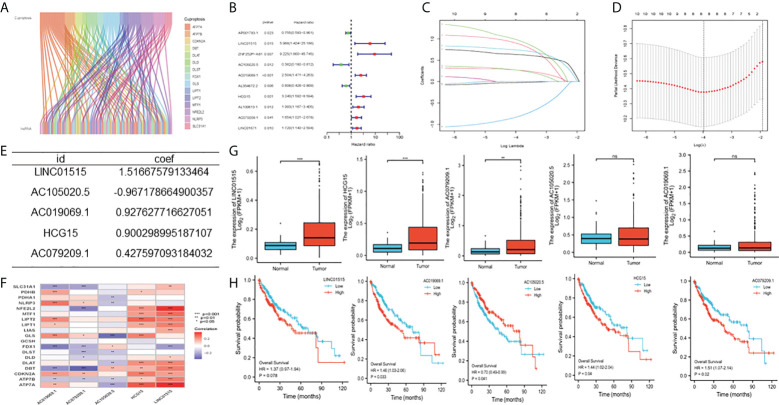
Construction of cuproptosis-related characteristics m TCGA-LIHC cohort **(A)** Co-expression analysis of cuproptosis related locRNAs, **(B)** Univariate Cox regression analysis **(C, D)** Lasso Cox regression analysis (lasso Lambda and lasso Cvfit), **(E)** Multivariate Cox regression analysis **(F)** Correlation between IncRNAs involved in model construction and cuproptosis-related genes **(G)** Expression level of five CDIs contained in signature **(H)** Kaplan-Meier (K-M) analyses OS based on the expression level of five CDIs TCGA, The Cancer Genome Atlas, CRLs, Cuproptosis related IncRNAs, OS overall survival. **Means P<0.01, ***means P<0.001, ns means no significance.

By comparing 373 HCC tumors and 51 normal tissues, we analyzed differentially expressed cuproptosis-related genes (**Supplementary Table 1**) in HCC and the expression of 53 CRLs (**Figure 1A**). We detected cuproptosis-related genes through the Human Protein Atlas (HPA) database (**Supplementary Figure 2**). Then, 10 CRLs with significant differential expression were obtained by univariate regression analysis (**Figure 1B**), Lasso Cox regression (**Figures 1C, D**), and multivariate Cox regression analysis. Five candidate OS-related CRLs were identified in the TCGA-LIHC cohort to find the best CRLs for establishing prognostic characteristics. Finally, the five key CRLs were extracted to construct the signature, which include LINC01515 and AC105020.5, AC019069.1, HCG15, and AC079209.1 (**Figure 1E**) showing the coexpression of cuproptosis-related genes (**Figure 1F**). Furthermore, we presented the box plots of expression levels (**Figure 1G)** and K-M curves of OS (**Figure 1H**) in the training set to study the expression levels and independent prognostic power of each characteristic CRLs. From the results, we found that the expression levels of LINC01515, HCG15, and AC079209.1 were significantly increased, while the expression levels of AC105020.5 and AC019069.1 were not significantly different between normal samples and HCC samples. In the separation K-M analysis of OS, AC105020.5, AC019069.1, HCG15, and AC079209.1 high-risk and low-risk groups’ survival had a significant difference, while LINC01515 was not statistically significant.

### Construct the prognostic characteristics of cuproptosis in patients with hepatocellular carcinoma

**Figure 2:**
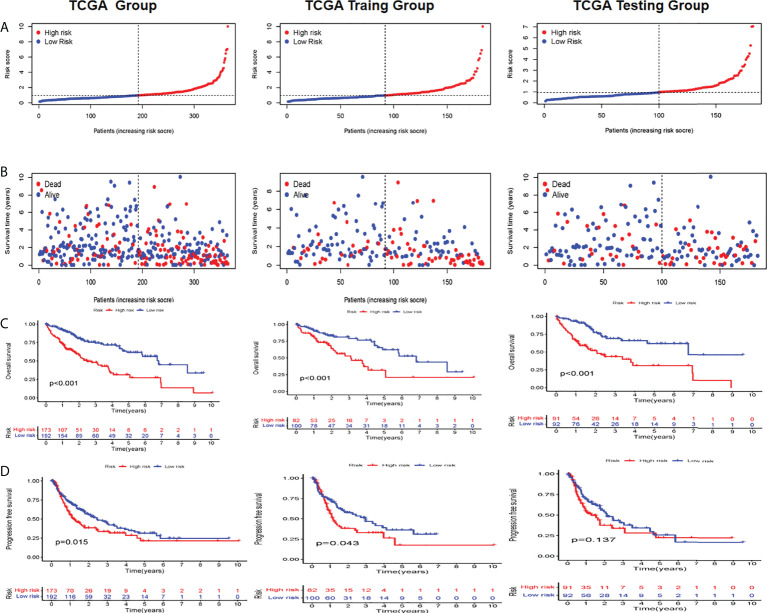
Evaluation and validation of the utility of CRLs in TCGA, training, and testing sets **(A)** Distribution of the patients normalized CRL scores **(B)** Patients’ overall survival (OS) time along with their CRL score **(C)** K M analyses of OS between high- and low-CRL groups **(D)** K-M analyses of PFS between high- and low CRI. groups in the training set. C1 cuproptosis index, CRL suproptons related lncRNAs, K M Kaplan-Meier, OS overall survival PFS, progression-free survival.

Based on patient traits, we calculated the CI for each patient below: CI = expression of LINC01515 * 1.516676 − expression of AC1050205 * 0.967179 + AC0190691 expression * 0.927628 + HCG15 expression * 0.900299 + AC079209.1 * 0.427597. In addition, based on the CI median value, HCC patients in the training set can be separated into the high-CI group and low-CI group. The CI can be adjusted to make the data more direct (**Figure 2A**). In the TCGA-LIHC data set and compared to the low-CI group, the high-CI group patients had a higher rate of death (**Figure 2B**). To assess the prognostic feasibility of CI, a K-M analysis was performed to decipher that it can be seen that the high-CI group had a significantly lower OS than the low-CI group (**Figure 2C**). It was the same as the evaluation of PFS in the testing, training, and TCGA data set (**Figure 2D**).

### PCA analysis and comprehensive evaluation of clinical parameters of cuproptosis-related lncRNA model in patients with hepatocellular carcinoma

**Figure 3:**
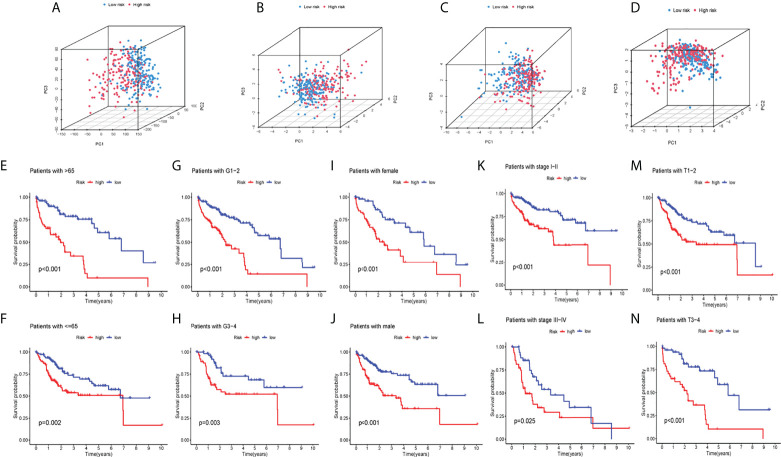
PCA analysis and systematic evaluation of CRLs and clinical parameters in HCC patients. **(A)** allGene PCA analysis; **(B)** cuproptosisLncRNA PCA analysis; **(C)** cuproptosisGene PCA analysis; **(D)** riskLnc PCA analysis; **(E–N)** Survival analyses of the risk score in different subgroups of various clinical factors: age (≤/>65years), pathological grade (1–2/3–4), sex (female/male),clinical stage (I–II/III–IV) and pathological T(T1-2/3-4).

PCA analysis was carried out on all genes, cuproptosis-related genes, cuproptosis-related lncRNAs, and risk lncRNAs of the model (**Figures 3A–D**), and the CRLs’ clinical availability was further clarified. The lncRNAs involved in the model construction can effectively distinguish high-risk group patients and low-risk patients, proving the model’s accuracy. The correlation between CI and clinical traits was further established, and the CI validity in predicting other clinical parameters was improved. Different levels of different clinical parameters (including age, sex, clinical stage, clinical grade, and pathological T) of the training set had remarkable deviations in adjusted CI (*P*< 0.05). Higher CI may be related to late clinical-stage, stage, and pathological T stage (**Figures 3E–N**), thus proving that our model department is used to distinguish patients with different clinical–pathological characteristics.

### Development and evaluation of cuproptosis-related clinicopathological nomogram

**Figure 4:**
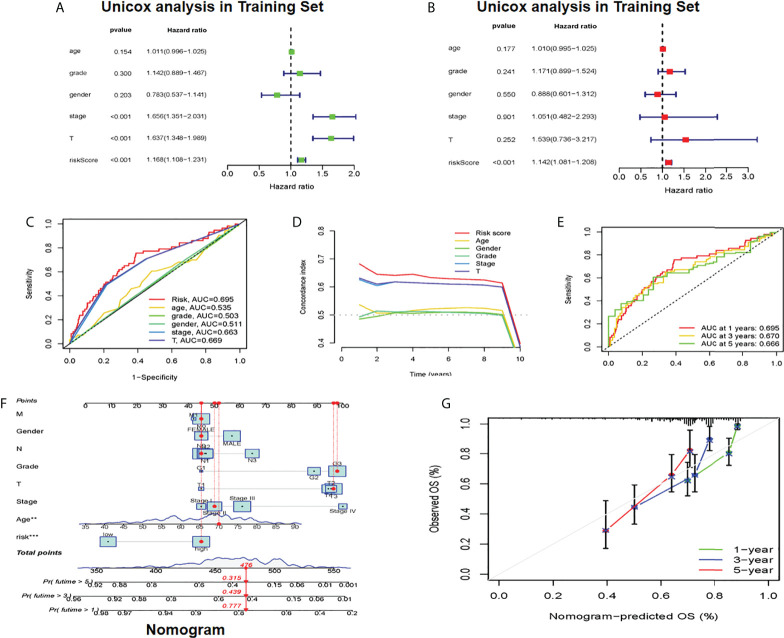
Establishment of cuproptosis-related clinicopathologic nomogram **(A)** Univariate Cox regression analysts of the signature CRLs and clinical parameters **(B)** Multivariate Cox regression analysis of the signature CRLs and clinical parameters **(C)** Development of a prognostic nomogram to predict 1-, 3- and 5-year OS in the HCC patients of the training set. **(D)** Clinical data c-index ROC curve **(E)** Characteristic CRLs to predict 1-, 3- and 5-OS of HCC patients in the traming set. **(F)** A prognostic nomogram was developed to predict the 1-, 3- and 5-year OS of HCC patients in the training set. **(G)** Prognostic nomogram predicted OS time, CRLs, cuproptosis-related lncRNAs; OS overall survival, HCC, hepatocellular carcinoma. *Means P<0.05, **Means P<0.01, ***means P<0.001.

Whether it was an independent prognostic indicator explored by conducting univariate and multivariate Cox regression analyses in the training set (**Figures 4A, B**). It was found that the age, risk score, and OS of HCC patients were markedly related in univariate and multivariate Cox analyses. The clinical ROC curve assessed the accuracy with an AUC value of 0.695 (**Figure 4C**). The result of the c-index curve is the same as the former (**Figure 4D**). In addition, the ROC curve had good results in evaluating the 1-, 3- and 5-year survival of patients (**Figure 4E**). Based on the above results, individual 1-, 3-, and 5-year OS was predicted by clinicopathological nomogram advance (**Figure 4F**). We performed survival descriptions on the calibration plots to confirm that the nomogram predictions were identified as satisfactory and the predictions were great (**Figure 4G**). In conclusion, multiple aspects illustrated the validity of prognostic maps.

### Gene ontology function of risk differential genes and gene set variation analysis pathway analysis

**Figure 5:**
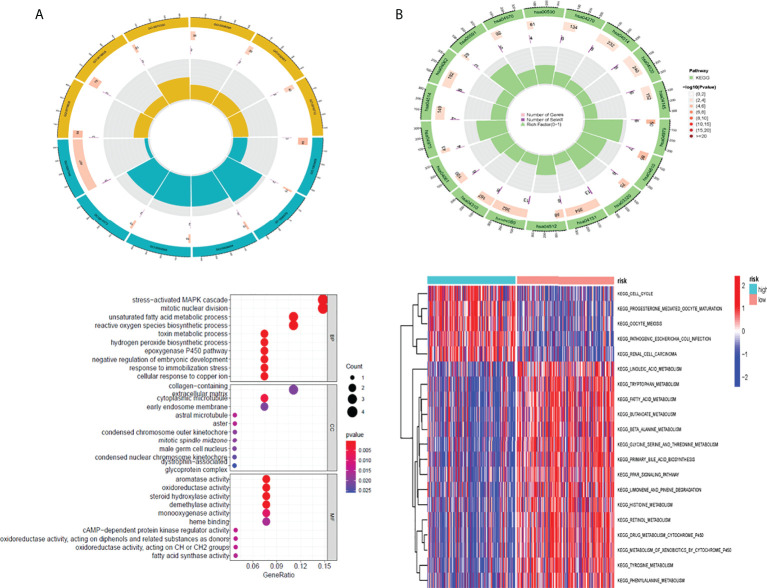
The result of GO and GSVA pathway enrichment analyses. The result of GO and GSVA pathway enrichment analyses **(A)** Biological process, cellular component, molecular function GO terms for risk DEGs **(B)** GSVA pathways for DEGs The top 10 sorted by the GeneRatio of GO terms and the top 20 sorted by GSVA pathways are shown. GO, Gene Ontology; GSVA, gene set variation analysis; DEGs, differentially expressed genes.

First, we analyzed the difference of genes in high-risk and low-risk groups and found that the risk difference genes are based on their mean value in high-risk and low-risk groups (**Supplementary Table 2**). We then used the “clusterprofiler” software package to conduct enrichment analysis on differentially expressed genes (DEGs) to explore their biological characteristics (30). Biological process (BP) terminology indicates that DEGs are rich in the “emphasizing-activating MAPK cascade,” “toxin metabolic process,” and “cyclooxygenase P450 pathway.” In terms of cell composition (CC), “glycoprotein complex” “astral microtubule” and “cytoplasmic microtubule” are significantly abundant. Therefore, we hypothesized that DEGs mainly play a role in the extracellular matrix. The main enrichment molecular function (MF) terms of DEGs are “oxidoreductase activity” “heme binding” and “sulfur compounds” (**Figure 5A**).

In addition, this study utilized the “clusterprofiler” software package for GSVA path enrichment analysis (30) for studying further roles of DEGs. According to the results, the following pathways were significantly enriched in DEGs: “cell cycle”, “fatty acid metabolism” and “drug metabolism cytochrome P450” (**Figure 5B**). In conclusion, the results confirm that these central genes are involved in cellular metabolic processes.

### Landscape of the hepatocellular carcinoma mutation profiles and survival analysis

**Figure 6:**
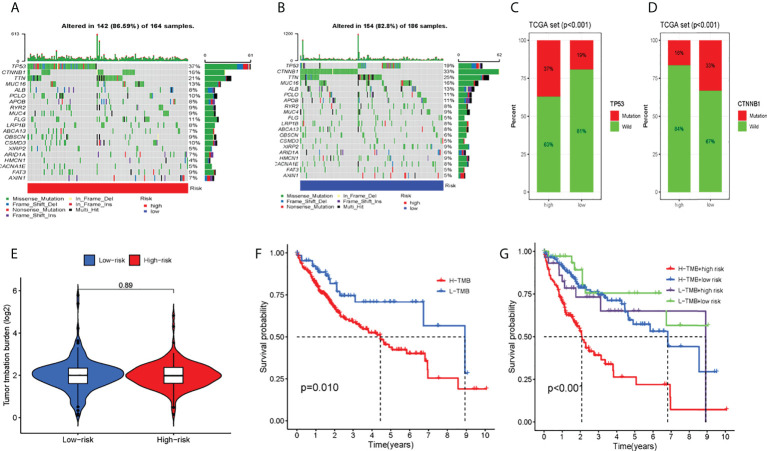
Tumor somatic mutation and differential tumor mutational burden (TMB) and survival analysis. The waterfall plot of tumor somatic mutation was established by those with high-risk scores **(A)** and low-risk scores **(B)**. **(C)** TP53 mutation, **(D)** CTNMBI mutation **(E)** Analysis of the difference of TMB; **(F)**TMB survival analysis; **(G)** Combined survival analysis of TMB and patient risk; Each column represented individual patients. The upper barplot showed TMB. The number on the right indicated the mutation frequency in each gene The right barplot showed the proportion of each variant type. TMB tumor mutational burden.

In total, this study explored the somatic mutation spectrum of 371 HCC patients in a VCF format through the “maftools” software package (29) and selected the 20 genes with the highest mutation frequency for visualization. The waterfall plot showed that the first three mutated genes were TP53, CTNNB1, and TTN mutations in HCC samples. We not only counted the number of variants in each sample but also marked the HCC mutation kinds in box plots in different colors. Compared with the low-risk group, most high-risk group genes had higher mutation frequencies (**Figures 6A, B**). For TP53 and CTNNB1 with high mutation frequency, we found that the wild-type frequency in the high-risk and low-risk groups was higher than that of the mutant, and the difference was statistically significant (**Figures 6C, D**). In addition, we assessed TMB in both groups, meanwhile. We found that the high-risk group's TMB was not greatly different from that of the low-risk group (P = 0.89) (Figure 6E). Between the high and low mutation burden groups, however, there was a big difference in patient survival (P = 0.010). Compared with the high mutational burden group, patients in the low mutational burden group had a better prognosis (**Figures 6F**). Combining TMB with the risk score of patients, the survival rate of the four groups was also significantly different (P < 0.001) (**Figures 6G**).

### Potential significance of immunotherapy based on characteristics and tumor immune microenvironment landscape estimation

**Figure 7:**
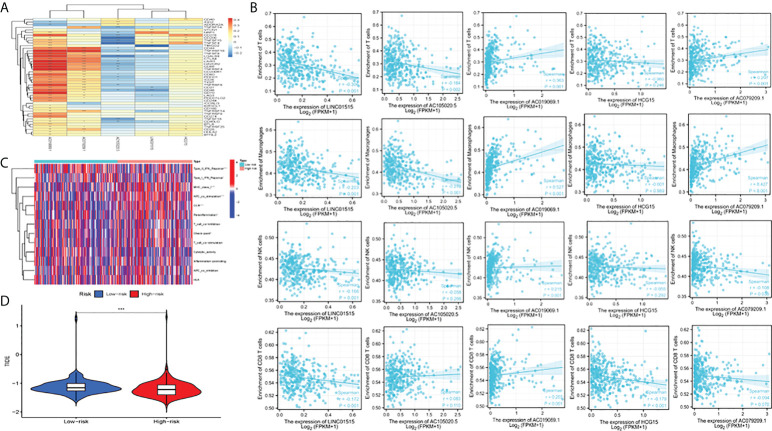
Analysis of immune function, escape, and immunotherapy. **(A)** Correlation analysis between CRLs and the immune checkpoint; **(B)** Correlation analysis between CRLs and immune cells (T cells, macrophage cells, NK cells and CD8 T cells); **(C)** Analysis of correlation and difference between high-risk and low-risk groups and immune function; **(D)** TIDE between high and low-risk groups; TIDE, tumor immune dysfunction and exclusion. *Means P<0.05, **Means P<0.01, ***means P<0.001.

We assessed the association of five CRLs with immune checkpoints (35) using the Wilcoxon test (**Figure 7A**) and found that they were significantly associated with PD1 (PDCD1), PDL1 (CD274), CTLA4, and other common immune checkpoints. This is consistent with the Shapiro–Wilk Normality test (**Supplementary Figure 3**). In addition, we measured the content of four tumor immune-infiltrating cells (T cells, macrophages, NK cells, and CD8 T cells) in the gene expression matrix in the TCGA-LIHC cohort of HCC samples by using ssGSEA analysis. The scatter diagram was used to reveal the results of the immunoanalysis. Most of the results were statistically significant (*P*< 0.05) (**Figure 7B**). Additionally, we discussed the relationship between CRL characteristics and immune function in HCC. According to the heat map, some parameters were significantly different between high-risk and low-risk groups, such as APC_co_Stimulation, Type_I_IFN_Response, MHC_class_I, Type_II_IFN_Response, and CCR (**Figure 7C**). Finally, the effect of immunotherapy in high-risk and low-risk patients was assessed by assessing differences in immune escape and immunotherapy in high-risk and low-risk groups (**Figure 7D**). It was found that low-risk group TIDE (tumor immune dysfunction and exclusion) was higher, indicating that the greater the potential of immune escape, the worse the effect of immunotherapy (*P*< 0.001).

### Screening potential drugs for hepatocellular carcinoma

**Figure 8:**
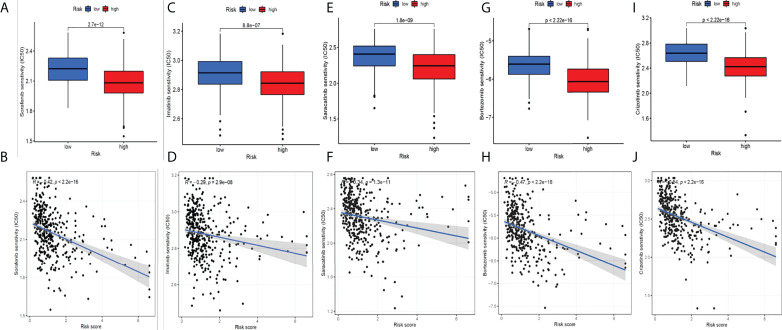
Screening potential drugs for HCC. Five drugs with great differences in drug sensitivity between two groups: sorafenib **(A-B)**, imatinib **(C-D)**, saracatinib **(E-F)**, bortezomib **(G-H)** and crizotinib **(I-J)**.

We circulated the drugs and observed which drugs had different sensitivities between the two groups. The lower the IC50 value, the higher the sensitivity to the drugs. Finally, we screened five drugs with great differences in drug sensitivity between two groups, including sorafenib (**Figures 8A, B**), imatinib (**Figures 8C, D**), and saracatinib (**Figures 8E, F**), bortezomib (**Figures 8G, H**), and crizotinib (**Figures 8I, J**). In conclusion, this provides a great reference for clinical medication.

## Discussion

HCC has a high recurrence rate and is one of the leading causes of tumor-related deaths. The prognosis of patients is seriously affected by the high recurrence rate, and there is currently no effective preventive method. With the rapid development of systemic therapy, after sorafenib treatment, more and more drugs are available but survival-enhancing treatments remain unsatisfactory (36). Moreover, HCC treatments in recent years utilized the immunotherapy of immune checkpoints (32), but the therapeutic effect is not very ideal. Therefore, it is urgent to explore the occurrence, development, recurrence, migration, and HCC immunotherapy mechanism. Xing et al. have reported the diagnostic and prognostic value of genes related to the focal degeneration of HCC (37). In this way, key regulatory pathways or networks in HCC are further revealed, and the development and improvement of related therapeutic approaches are facilitated.

Recently, cuproptosis has been considered as a copper-triggered mode of mitochondrial cell death (9). In the case of the growth and severity of cancer, it was reported that Cu might exert an important function (33, 34). Related research supported this hypothesis. For instance, liver cancer in patients with Wilson’s disease had an increased incidence. The relation between staging and Cu levels in colorectal cancer and breast cancer, Cu exposure, and the relation between pancreatic cancer and prostate cancer have also been observed (37–40). Studies have shown that the demand for NET (neutrophil extracellular trap) formation by different trace elements varies greatly; unlike zinc, low or negligible copper levels will not interfere with the NET formation and may even enhance NET formation. In contrast, high copper concentrations inhibit net release, but this was mainly due to cytotoxicity to neutrophils (41). Several mechanisms of copper-dependent tumor growth and development have been studied in recent years (8, 42, 43). Tsvetkov P et al. showed that the mechanism of copper-dependent regulation of cell death is different from the known death mechanism and depends on mitochondrial respiration. Copper-dependent death occurs through the direct combination of copper and the fatty acylation component of the tricarboxylic acid (TCA) cycle. This leads to the aggregation of acylated proteins and the subsequent loss of iron–sulfur cluster proteins, which lead to protein toxic stress and eventually cell death. These findings can explain the necessity of the steady-state mechanism of ancient copper (44). In addition, Cu can also promote angiogenesis, which is very important in tumor metastasis. In particular, more and more lines of evidence showed that several angiogenic factors could be stimulated by copper, including the vascular endothelial growth factor (VEGF), angiopoietin (hAng), and interleukin-1 (IL-1) (42, 45, 46). Additionally, Yang et al. found that the COMMD 10-inhibited HIF1α/CP loop can enhance the iron wire disease and radiosensitivity by destroying the Cu-Fe homeostasis in HCC. This work provided a new target and treatment strategy for overcoming the radioresistance of HCC (47). Our results were mostly consistent with most of the DEGs in their research. We, however, collected more DEGs because we registered more lncRNAs related to cuproptosis by reviewing the latest literature. It was found that cuproptosis studies are developing rapidly, and more and more discoveries are being revealed.

Based on the key role of cuproptosis in cancer and the close interaction between cuproptosis and lncRNA, we used TCGA transcriptome data to study the potential mechanism and prognostic value of CRLs in HCC. We identified five key CRLs (LINC01515, AC105020.5, AC019069.1, HCG15, AC079209.1). They were applied to develop a risk-scoring model. In this way, a patient’s prognosis could be differentiated. Importantly, an independent prognostic HCC factor was the risk model. A prognostic nomogram with high precision was next established to provide 1-, 3-, and 5-year HCC OS prediction. This greatly improved the feasibility of CRLs in judging the prognosis of patients. Moreover, there was a significant correlation between CRLs and the immune-related function, immune escape, and immunotherapy of HCC. Given the essential role of the TME in tumorigenesis and development, the interaction between cancer cells and immune cells regulates all links to tumor development. As a result, CRL-mediated variations may affect tumor progression (48) through immune-related mechanisms. In this study, we investigated that the expression of the immune checkpoint, containing PD-L1, PD-1, and CTLA-4, and five CRLs were significantly correlated with the above immune checkpoints. These findings suggest the potential role of CRLs in regulating immune checkpoint expression in the TME. In addition, differences between high- and low-risk groups in immune escape and immunotherapy were also assessed, and the influence of immunotherapy on high- and low-risk patients was then assessed. The results showed that the TIDE of the low-risk group was higher, and the greater the potential of immune escape in the low-risk group, the worse the effect of immune treatment. Based on this, we also screened the model-based sensitivity analysis of patients to chemotherapy drugs. Five drugs with significant expression differences were extracted, namely sorafenib, imatinib, saracatinib, bortezomib, and crizotinib, which provided a reliable choice for clinical medication.

A large number of studies have shown that lncRNA plays a very important role in the TME. For instance, Huang et al. showed that lncRNA can stimulate the differentiation of T regulatory cells, promote the immune escape of HCC cells, and can be used as a diagnostic biomarker of HCC (49). As for the five key CRLs, numerous studies explored the LINC01515 function and HCG15 function in cancer. Liu (50) et al. found that the expression of LINC01515 was increased in nasopharyngeal carcinoma, and the higher the expression of LINC01515, the worse its prognosis. This agreed with the findings of this study. In addition, earlier research (51) suggested that LINC01515 exerted a key function in the drug resistance of leukemia cells. However, in our study, LINC01515 was identified as the CRLs of HCC, while Liu reported lncRNA-related immunity. It was found in tumors that lncRNA had a complicated role. Yan (52) et al. found that HCG15 is a hypoxia-reactive lncRNA that improved HCC cell propagation and aggression by enhancing ZNF641 transcription. In addition, in the regulation of glioma formation, the PABPC5/HCG15/ZNF331 feedback loop involving HCG15 exerted a significant function, giving a novel target for glioma therapy (53). Additionally, the prognostic model involved in HCG15 is conducive to discovering the new mechanism of ivermectin-inhibiting ovarian cancer cells and the benefits of ivermectin-related molecular combination changes on its prediction in ovarian cancer, personalized drug treatment, and the prognostic evaluation of preventive and personalized drugs (PPPM) (54). Therefore, additional research on these newly discovered lncRNAs is essential.

There are some limitations on this study. First, the findings need to be further verified experimentally because the data and results of this study were based on TCGA transcriptomic, mutational, and clinical data. Then, the training and testing group were randomly grouped from the TCGA queue, which needs to be further verified by other databases.

## Conclusion

In conclusion, a new model based on CRLs has been developed, which has an important potential to forecast HCC prognosis. This study is expected to give a novel perspective on the underlying mechanisms of CRLs in regulating the immune microenvironment and immunotherapy.

## Data availability statement

The datasets presented in this study can be found in online repositories. The names of the repository/repositories and accession number(s) can be found in the article/**Supplementary Material**.

## Ethics statement

Consent from all participants was obtained through The Cancer Genome Atlas (TCGA).

## Author contributions

All authors participated in the present study, including conception and design (SC and PL), data collection (PH, JeL, LZ and HY), data analysis (SC and PL), drafting the article or critically revising (JaL) and study supervision (JaL). All authors contributed to the article and approved the submitted version.

## Funding

This work was funded by the Natural Science Foundation of Tianjin City (20JCYBJC01150); Tianjin Health Science and Technology Project (No. TJWJ2021QN063, No. TJWJ2021ZD010 and No. TJWJ2021MS034) and Tianjin Key Medical Discipline (Specialty) Construction Project.

## Acknowledgments

The authors are grateful to the contributors to the public databases used in this study and the reviewers for their constructive and helpful comments.

## Conflict of interest

The authors declare that the research was conducted in the absence of any commercial or financial relationships that could be construed as a potential conflict of interest.

## Publisher’s note

All claims expressed in this article are solely those of the authors and do not necessarily represent those of their affiliated organizations, or those of the publisher, the editors and the reviewers. Any product that may be evaluated in this article, or claim that may be made by its manufacturer, is not guaranteed or endorsed by the publisher.

## Supplementary material

Supplementary Figure 1 Flowchart of the data analysis procedures.

Supplementary Figure 2 Immunohistochemical staining of genes related to cuproptosis.

Supplementary Figure 3 Correlation analysis between CRLs and common immune checkpoints.

Supplementary Table 1 Identification of differentially expressed cuproptosis-related genes in HCC.

Supplementary Table 2 Screening of genes with different risks of cuproptosis.
