# Supplementary figures and images for: A novel cuproptosis-related prognostic lncRNA signature for predicting immune and drug therapy response in hepatocellular carcinoma
Source: Front Immunol. 2022 Sep 15;13:954653. doi: 10.3389/fimmu.2022.954653 (PMC9521313; doi:10.3389/fimmu.2022.954653)

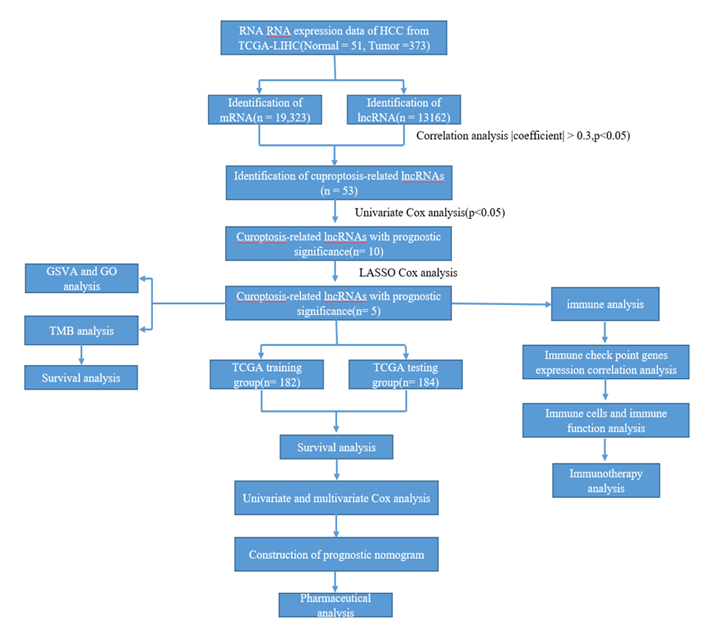

Supplement: Supplementary Figure 1 — Flowchart of the data analysis procedures. [file Image_1.png]

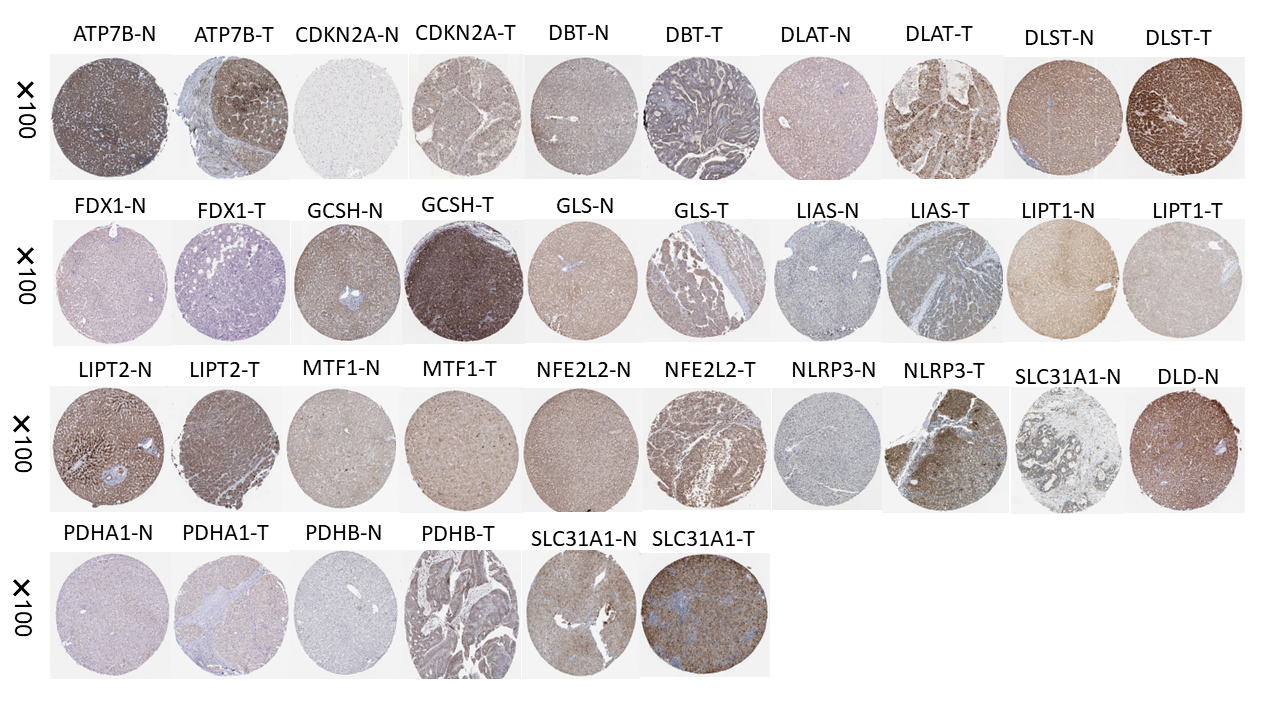

Supplement: Supplementary Figure 2 — Immunohistochemical staining of genes related to cuproptosis. [file Image_2.tif]

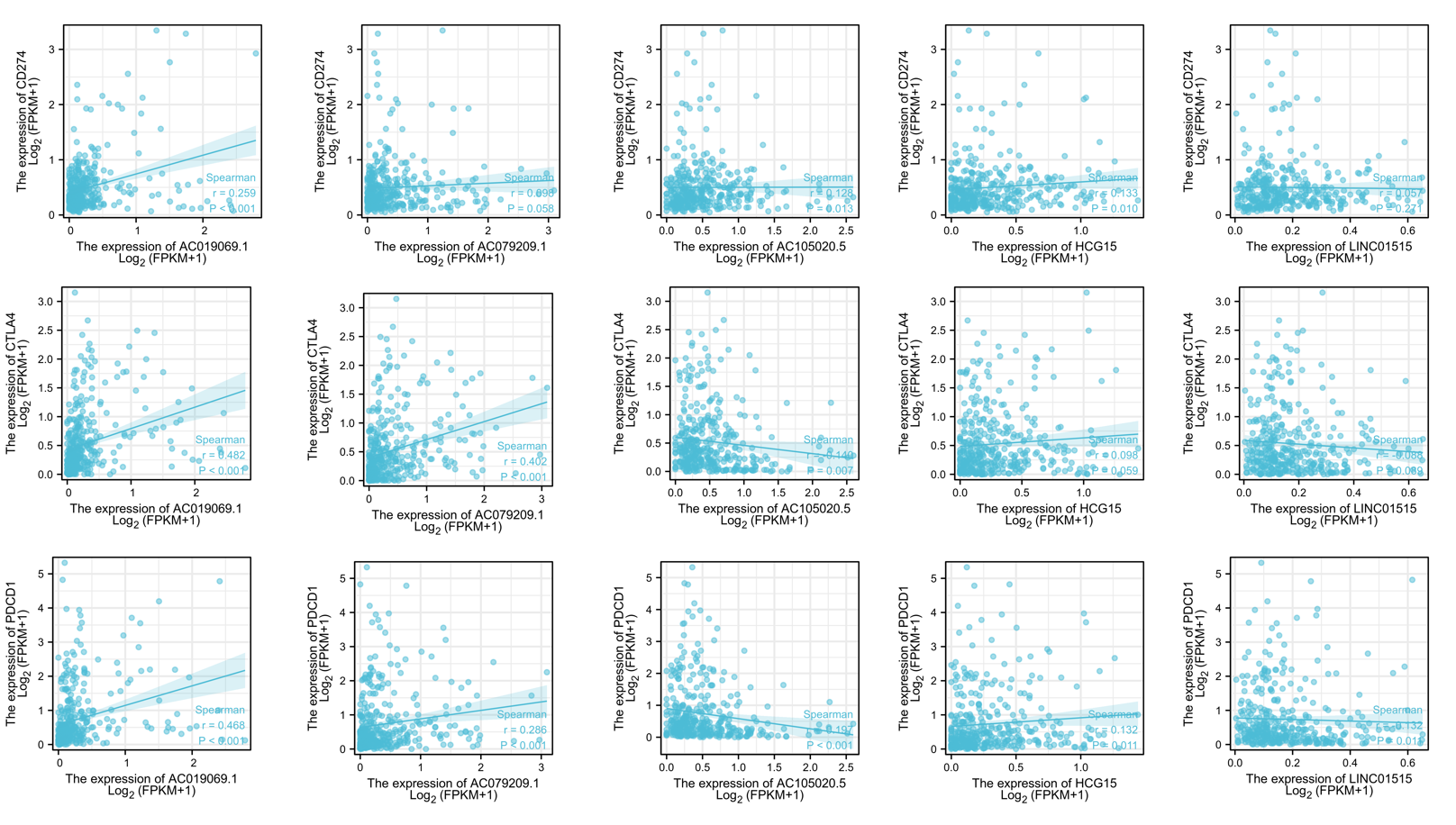

Supplement: Supplementary Figure 3 — Correlation analysis between CRLs and common immune checkpoints. [file Image_3.tif]
